# Controlled Formation of Polyimide Aerogel Networks in Carbon Fiber Felt via Multicycle Freeze-Drying for Thermal Protection

**DOI:** 10.3390/polym18060742

**Published:** 2026-03-18

**Authors:** Jae Won Lee, Han Kim, Yong-Ho Choa, Sook Young Moon

**Affiliations:** 1 Institute of Advanced Composite Materials, Korea Institute of Science and Technology (KIST), Chudong-Ro 92, Bongdong-Eup, Wanju-Gun 55324, Jeonbuk, Republic of Korea; 2 Department of Materials Science and Chemical Engineering, Hanyang University, Ansan-si 15588, Gyeonggi-do, Republic of Korea

**Keywords:** carbon fiber/polyimide aerogel composites, multicycle impregnation, polyimide-derived char layer, microstructure control, thermal protection

## Abstract

Fiber-reinforced aerogel composites are attractive for thermal protection applications because porous polymer networks can suppress heat transfer while maintaining structural stability. In this study, carbon fiber felt was integrated with a polyimide aerogel via a freeze-drying-assisted multicycle impregnation process to achieve controlled formation of interconnected aerogel networks within the fibrous scaffold. With increasing impregnation cycles, the composites exhibited progressive microstructural densification and improved structural stability. Although bulk density increased, thermal protection performance under prolonged butane-torch exposure was significantly enhanced, showing delayed backside temperature rise and improved resistance to structural degradation compared with bare carbon felt. Post-ablation analyses revealed the formation of a micro-/nanoporous polymer-derived char layer and a multilayer thermal-resistance structure, which contributed to suppressed heat transfer during flame exposure. These results indicate that effective thermal protection in CF/PA composites is governed by dynamic microstructural evolution and char-layer formation rather than intrinsic room-temperature thermal conductivity alone. The proposed multicycle impregnation strategy provides a scalable approach for designing lightweight polymer-based thermal protection materials operating in high-temperature environments.

## 1. Introduction

Polymer-based materials exposed to extreme thermal environments undergo complex heat transfer and degradation processes that critically determine their insulation performance and structural integrity [1,2,3]. In high-temperature applications, thermal protection strategies can be broadly divided into active systems employing regenerative cooling and passive systems that rely on controlled polymer pyrolysis, such as ablative materials [4,5]. In passive thermal protection, heat attenuation is achieved through endothermic decomposition reactions and the evolution of gaseous products, which collectively reduce heat flux and delay heat transfer to the underlying structure. These ablation processes are governed by strong coupling among thermomechanical, thermochemical, and thermophysical phenomena within the polymer matrix and its degradation products [6,7,8].

Polymer matrix composites have been extensively investigated as passive thermal protection materials owing to their thermal resistance, structural stability, and processing versatility. Among them, phenolic resin (PR)-based composites have served as representative systems because of their chemical stability and high-temperature resistance. However, the dense crosslinked polymer networks inherent to PR result in relatively high density and thermal conductivity, which limit further weight reduction and thermal insulation efficiency. To address these limitations, PR aerogels have been explored to introduce porous architectures and lower bulk density. Despite these advantages, challenges associated with microstructural controllability and insufficient oxidation resistance at elevated temperatures have restricted their applicability in demanding thermal environments [9,10,11,12,13,14,15].

In this context, polyimide aerogels (PAs) have emerged as promising polymer-based thermal insulation materials due to their intrinsic oxidation resistance, high thermal stability, and mechanical robustness at elevated temperatures [16,17,18]. From a polymer chemistry and physics perspective, the rigid aromatic backbone and stable imide linkages of polyimides contribute to the enhanced thermal durability and char-forming capability compared to phenolic systems. Previous studies have demonstrated that PA-based composites can achieve improved thermal insulation and mechanical performance through reinforcement with inorganic fibers and through tailored microstructural control strategies [19,20]. Among various reinforcement architectures, porous carbon fiber felt (CF) has been identified as an effective fibrous scaffold for aerogel integration, providing mechanical reinforcement while preserving low density and low thermal conductivity owing to its high porosity, dimensional stability, and cost effectiveness [21,22,23].

In the fabrication of carbon fiber/polyimide aerogel (CF/PA) composites, achieving uniform and sufficient impregnation of PA within the CF network remains a critical challenge, as impregnation quality directly governs composite density, mechanical integrity, thermal insulation performance, and char-layer evolution during thermal exposure [24,25,26]. PA-based composites are commonly fabricated using sol–gel processing, powder foaming, and freeze-drying techniques; however, each method exhibits inherent limitations associated with processing complexity, microstructural controllability, and volumetric shrinkage [27,28,29,30]. Although freeze-drying is effective for generating highly porous aerogel structures by suppressing capillary collapse during solvent removal, maintaining homogeneous PA distribution throughout the three-dimensional carbon fiber felt remains difficult [31,32,33,34,35,36,37,38]. In particular, non-uniform pore filling and localized shrinkage can occur during subsequent thermal imidization, leading to microstructural heterogeneity and compromised composite performance.

In this study, CF/PA composites were fabricated using a freeze-drying-assisted multicycle impregnation strategy to enhance PA distribution uniformity and structural stability within the carbon fiber felt. Repeated impregnation and freeze-drying enabled the gradual construction of interconnected PA networks throughout the internal pore structure, thereby mitigating volumetric shrinkage during thermal imidization and subsequent high-temperature exposure. Under thermal loading, polymer-derived char-layer formation is expected to play a key role in thermal protection, which is evaluated through butane-torch testing. Accordingly, the design concept in this work differs from conventional aerogel insulation approaches that mainly emphasize low intrinsic thermal conductivity. Instead, the present study focuses on dynamic thermal protection under flame exposure, where structural evolution and char formation dominate heat-transfer behavior. We hypothesize that repeated freeze-drying-assisted impregnation promotes progressive formation of interconnected polyimide networks within the carbon fiber scaffold. This microstructural evolution is expected to facilitate stable char-layer development during flame exposure and thereby enhance thermal protection performance.

Based on this, this work aims to systematically elucidate how a multicycle impregnation process governs microstructural evolution, pore architecture, and thermal-response behavior in CF/PA composites, providing polymer-processing-based insights into lightweight thermal protection behavior under localized high-temperature exposure.

## 2. Materials and Methods

### 2.1. Fabrication of CF/PA Composites

Poly (amic acid) (PAA) was synthesized by dissolving 9.180 g of 4,4′-oxydianiline (ODA, 97%, Sigma-Aldrich, Saint Louis, MO, USA) in 200 mL of N,N-dimethylformamide (DMF, 99%, Samchun Chemical Co., Ltd., Seoul, Republic of Korea) under continuous stirring, followed by the gradual addition of 10 g of pyromellitic dianhydride (PMDA, 97%, Sigma-Aldrich). The reaction mixture was stirred for 12 h to obtain a homogeneous PAA solution. Although the molecular weight of PAA was not directly measured, identical polymerization and processing conditions were maintained throughout the study to ensure reproducible solution characteristics suitable for impregnation. The resulting polymer was isolated by repeated precipitation in deionized water and thoroughly washed to remove residual solvent and unreacted species. Through this precipitation and washing process, residual DMF was effectively removed, and the obtained PAA powder was subsequently dried and used for the following aqueous processing step. All reagents and solvents were used as received without additional purification or distillation unless otherwise specified. For aqueous processing, 12 g of the dried PAA was dissolved in a mixed solvent consisting of 8 mL of triethylamine (TEA, >99.5%, Sigma-Aldrich) and 400 mL of distilled water under stirring for 5 h to obtain a clear poly (amic acid) salt (PAS) solution. The addition of TEA induced ionization of the carboxylic acid groups of PAA, forming ionic carboxylate–amine pairs and thereby enhancing polymer–water interactions, which enabled complete dissolution of PAS in the aqueous medium [39,40,41]. The resulting PAS solution was transferred to a rotary evaporator flask, and a piece of carbon fiber felt (rayon-based CF; density: 0.102 g/cm^3^; porosity: ~94%) with dimensions of 5 cm × 5 cm × 1 cm was fully immersed. The rayon-based CF consisted of randomly oriented carbon fibers forming a three-dimensional porous network, providing interconnected pathways for polymer infiltration and subsequent aerogel network formation. Solvent evaporation was carried out at 90 °C for 2 h to promote impregnation of the polymer solution into the porous CF network. The impregnated CF/PAS precursor was subsequently freeze-dried for 48 h and thermally imidized in a vacuum oven by sequential heat treatment at 100 °C, 200 °C, and 300 °C for 1 h at each temperature. This impregnation–freeze-drying–imidization sequence was repeated to control the amount and distribution of PA within the CF scaffold. The final composites were denoted as CF/PA-x, where x represents the number of fabrication cycles.

### 2.2. Characterization

The microstructures of the composites were examined using field-emission scanning electron microscopy (FE-SEM; Verios, FEI, Hillsboro, OR, USA) at an accelerating voltage of 10 kV using an ETD detector. Chemical bonding characteristics were analyzed by Fourier-transform infrared spectroscopy (FT-IR; iN10, Thermo Fisher Scientific, Waltham, MA, USA) in the range of 2000–600 cm^−1^ with a total collection time of 51 s. Structural features and carbon-related bonding were further investigated using Raman spectroscopy (InVia, Renishaw, Wotton-under-Edge, UK) with a 514 nm excitation laser. Raman spectra were collected from at least three representative points for each region, and the spectra shown represent representative results. Surface chemical states were analyzed by X-ray photoelectron spectroscopy (XPS; monochromatic Al Kα source, Thermo Fisher Scientific, USA). Thermal behavior was evaluated by thermogravimetry–differential scanning calorimetry (TG–DSC; Labsys Evo, Setaram, Plan-les-Ouates, Switzerland) under argon and oxygen atmospheres up to 1000 °C at a heating rate of 10 °C/min. TG–DSC measurements were performed using similar initial sample masses (approximately 10–15 mg) to ensure reliable comparison of weight-loss behavior. Thermal conductivity was measured using a transient plane source method (Hot Disk TPS 2500S, Hot Disk AB, Göteborg, Sweden) equipped with a Kapton sensor, operated at an output power of 10 mW for 10 s. Mechanical properties were measured using a universal testing machine (Model 4400, Instron, Norwood, MA, USA) in a three-point bending configuration with a load cell capacity of 2 kN and a crosshead speed of 2 mm/min. The thermal insulation performance under localized thermal shock was assessed using a butane torch test. Samples with dimensions of Φ30 mm × 8 mm were subjected to repeated torch exposure (300 s per cycle, 13 cycles in total) at a distance of 20 mm. The backside temperature was recorded at a fixed point located at the center of the sample backside using an infrared thermometer (testo 875-1i, Testo SE & Co., Titisee-Neustadt, Germany), while maintaining a constant measurement distance throughout the experiment. The measurement spot size followed the manufacturer’s optical specification. These characterization methods were combined to correlate microstructural evolution with thermal protection behavior under localized thermal loading. Textural properties including Brunauer–Emmett–Teller (BET) surface area and Barrett–Joyner–Halenda (BJH) pore size distribution of samples before and after the butane torch test were obtained from N_2_ adsorption–desorption isotherms measured at 77 K using a Surface Area and Pore Size Distribution Analyzer (BELSORP-max, MicrotracBEL Corp., Osaka, Japan). Prior to analysis, samples were degassed under vacuum at 100 °C for 6 h. The isotherm data were collected within a relative pressure (P/P_0_) range of 0.01–0.99.

## 3. Results and Discussion

### 3.1. Characterization of CF/PA Composites

**Figure 1:**
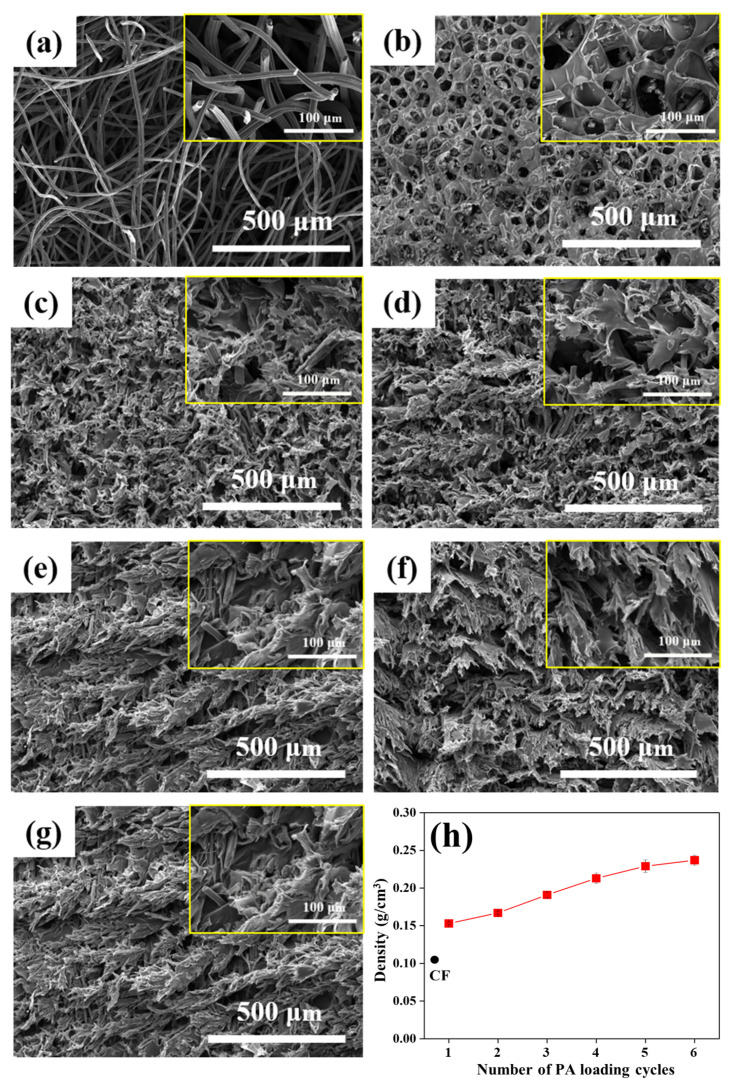
SEM images showing the microstructural evolution of CF/PA composites with increasing impregnation cycles: (**a**) CF, (**b**) CF/PA-1, (**c**) CF/PA-2, (**d**) CF/PA-3, (**e**) CF/PA-4, (**f**) CF/PA-5, and (**g**) CF/PA-6. (**h**) Corresponding density variation of the composites as a function of PA loading.

The microstructural evolution of CF/PA composites with increasing impregnation cycles was examined by SEM. Figure 1 presents cross-sectional SEM images of the bare CF and CF/PA-1 to CF/PA-6 composites. The bare CF exhibits a highly porous fibrous network composed of loosely interconnected fiber bundles and irregular void structures (Figure 1a). After a single impregnation cycle (CF/PA-1), partial filling of inter-fiber voids by the polymer phase is observed, forming localized polymer bridges while largely preserving the original porous framework (Figure 1b), indicating incomplete impregnation at the initial stage. With increasing impregnation cycles, progressive filling of the porous space and coverage of the CF skeleton by the polyimide phase are observed (Figure 1c–g), leading to gradual densification of the composite structure. In particular, the CF/PA-6 specimen exhibits substantially reduced pore boundaries and a continuous polymer phase surrounding the fiber bundles. The polymer film becomes thicker and more uniformly distributed along the fiber orientation, suggesting cumulative infiltration and consolidation of the polyimide matrix within the CF network. Consistent with these observations, the composite density systematically increases with impregnation cycle number (Figure 1h), reflecting reduced pore volume and expansion of the fiber–matrix interfacial region. These observations imply that repeated impregnation and imidization cycles promote progressive structural densification and interfacial evolution within the CF scaffold.

### 3.2. Mechanical Endurance of CF/PA Composites

**Figure 2:**
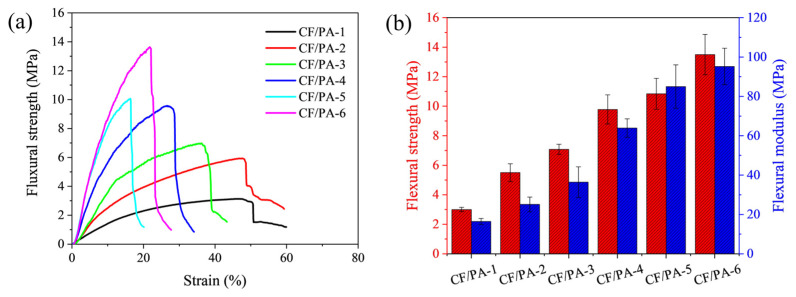
(**a**) Representative flexural stress–strain curves of CF/PA composites. (**b**) Flexural strength and flexural modulus as a function of PA impregnation cycles. Error bars represent standard deviation (n = 5).

Figure 2a presents the flexural stress–strain curves of CF/PA composites as a function of impregnation cycle number. For comparison, the flexural response of the bare carbon fiber felt is shown in Figure S1. The bare felt exhibits very low flexural stress with large apparent strain, reflecting deformation dominated by fiber rearrangement and network densification rather than intrinsic fiber elongation. The CF/PA-1 specimen shows a relatively flexible mechanical response, with a flexural strength of approximately 3.0 MPa and a flexural modulus of 15.6 MPa (Figure 2b), due to its low polyimide content and highly porous structure. With increasing impregnation cycles, progressive polymer infiltration and subsequent imidization lead to reduced porosity and improved fiber–matrix connectivity. As a result, both strength and stiffness increase systematically, reaching approximately 13.5 MPa and 95.2 MPa, respectively, for CF/PA-6. The stress–strain curves show a gradual transition toward stiffer behavior with reduced deformation after the maximum stress. At higher impregnation cycles (e.g., CF/PA-6), the abrupt post-peak stress drop indicates brittle failure associated with reduced structural compliance. Slight deviations from the monotonic trend (e.g., PA-2 and PA-5) are attributed to local variations in polymer infiltration and pore filling during intermediate stages. These changes in load-bearing behavior highlight how repeated impregnation progressively stabilizes the fiber network, providing a mechanically robust framework for subsequent thermal exposure.

### 3.3. Intrinsic Thermal Properties of CF/PA Composites

**Figure 3:**
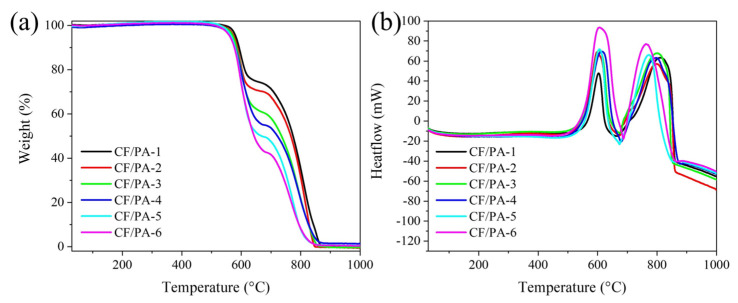
(**a**) TGA curves and (**b**) DSC curves of CF/PA composites, illustrating the thermal degradation and oxidation behavior of the composites.

Thermal stability under oxidative conditions is a key intrinsic property governing the high-temperature durability of polymer-based composite systems. To examine the thermo-oxidative behavior of the CF/PA composites, thermogravimetric analysis (TGA) and differential scanning calorimetry (DSC) were conducted under an oxygen atmosphere (Figure 3). The TG–DSC analysis in this study focuses on comparative thermo-oxidative degradation behavior rather than absolute residual mass, since the relative polymer and carbon fiber fractions vary with impregnation cycles. A slight apparent weight increase observed during the initial heating stage is attributed to baseline drift and/or minor oxygen uptake at reactive surface sites, which is commonly observed before the onset of active thermal decomposition. All CF/PA composites exhibit a characteristic two-stage mass-loss behavior (Figure 3a). The first mass-loss region, occurring at approximately 500–670 °C, is attributed to oxidative degradation of the polyimide matrix, accompanied by the evolution of volatile decomposition products such as CO, CO_2_, H_2_O, and CH_4_ [42]. The second mass-loss region, observed between 700 and 900 °C, corresponds to oxidation of the carbon fiber framework, resulting in near-complete mass loss at elevated temperatures. Compared with bare CF, the CF/PA composites show more gradual decomposition profiles, indicating that polymer incorporation modifies the oxidative reaction pathway within the composite structure. The DSC curves provide further insight into the decomposition process (Figure 3b). The first exothermic peak, associated with oxidation of the polyimide phase, appears in the temperature range of approximately 497–689 °C. The intensity of this peak increases with impregnation cycles, rising from ~50 mW for CF/PA-1 to ~90 mW for CF/PA-6 (~1.8×), reflecting increased polymer loading in the composites. In addition, slight variations in peak position and shape suggest differences in local thermal environments and interfacial interactions between the polymer matrix and carbon fiber network. The second decomposition stage involves concurrent oxidation of residual polymer-derived structures and carbon fibers, leading to progressive mass loss up to approximately 900 °C. These results indicated that repeated impregnation alters the thermo-oxidative decomposition behavior of CF/PA composites by modifying polymer content and interfacial thermal response within the composite structure.

### 3.4. Thermal Insulation Properties of CF/PA Composites (Revised)

**Figure 4:**
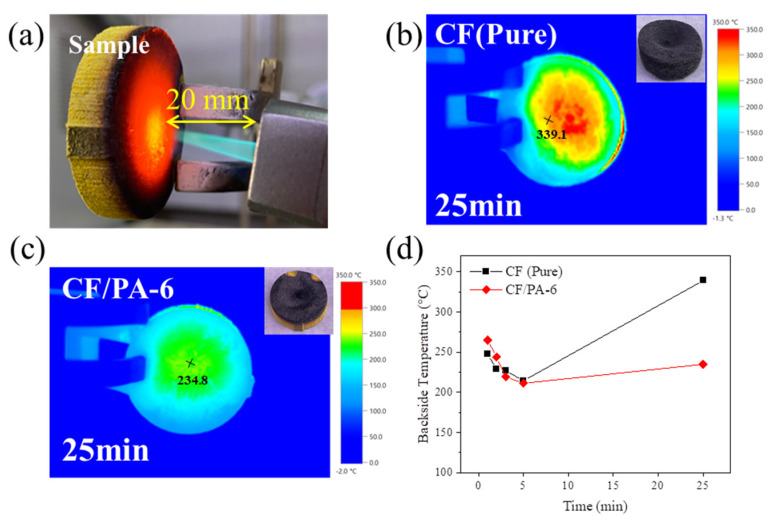
Ablation performance of CF and CF/PA-6 under repeated butane-torch exposure: (**a**) optical images of the ablation tests, infrared images of (**b**) CF and (**c**) CF/PA-6 during ablation at 25 min, and (**d**) backside temperature profiles as a function of exposure time.

Figure 4a presents the ablation test configuration and backside temperature evolution of bare CF and CF/PA composites during repeated butane-torch exposure. During the initial 5 min of exposure, both specimens maintained relatively stable backside temperatures. With prolonged thermal exposure, however, distinct differences in insulation behavior became evident. After 25 min (five cycles), the backside temperature of bare CF increased sharply from 214.4 °C to 339.1 °C, indicating rapid degradation of its thermal barrier function due to oxidation of the fiber network and microstructural collapse (Figure 4b). In contrast, CF/PA-6 exhibited a much more gradual temperature increase, rising from 211.2 °C to 234.8 °C over the same period (Figure 4c,d). Even after 50 min (10 cycles) and 65 min (13 cycles) of exposure, CF/PA-6 retained structural continuity with a backside temperature of 283.7 °C, whereas bare CF experienced severe erosion and through-thickness failure (Figure S2). The mass ablation rates summarized in Table 1 further confirm that CF/PA-6 maintains significantly lower mass loss than bare CF under prolonged high-temperature exposure. These results indicate that thermal insulation performance under flame exposure cannot be directly interpreted from room-temperature bulk thermal conductivity, highlighting the importance of structural evolution during ablation.

**Figure 5:**
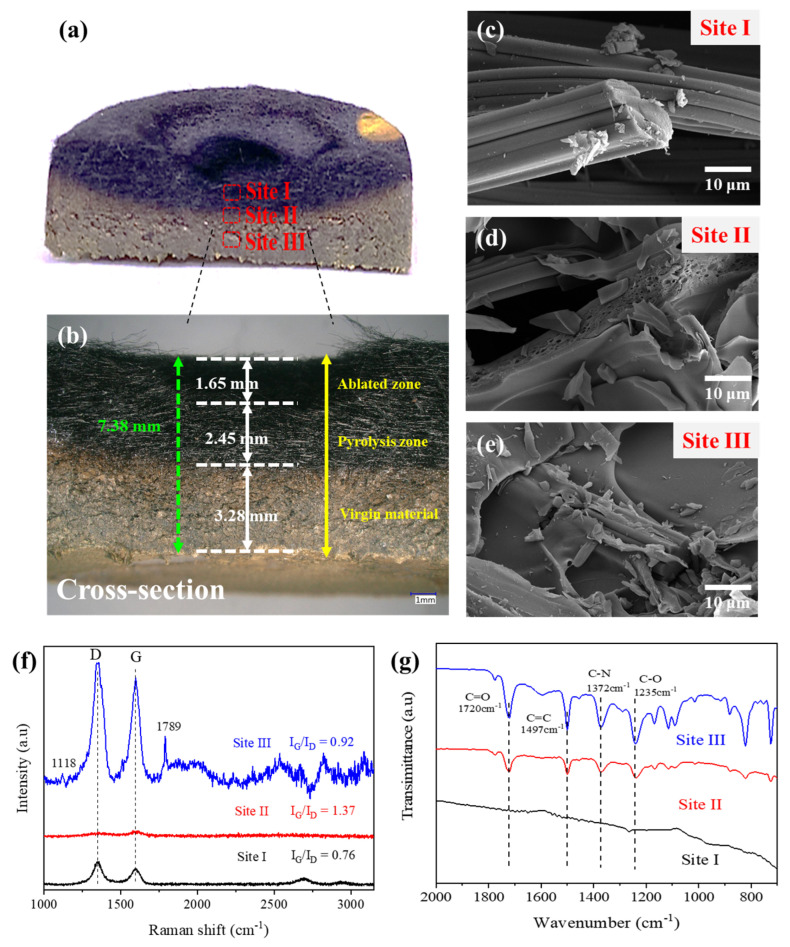
Cross-sectional characterization of CF/PA-6 after ablation: (**a**,**b**) cross-sectional images showing the ablated zone, pyrolysis zone, and underlying composite region; (**c**–**e**) SEM images collected at different depths from the exposed surface; (**f**) Raman spectra and (**g**) FT-IR spectra obtained from characteristic regions (Sites I–III). These results reveal the chemical and structural evolution of the composite during thermal exposure.

In conventional polymeric foams (e.g., polyisocyanurate foams), thermal insulation primarily relies on low intrinsic thermal conductivity under ambient conditions, whereas the present CF/PA composites are designed for thermal protection under prolonged flame exposure. In this case, dynamic char formation, structural stability, and resistance to erosion govern the effective thermal shielding performance [5], rather than bulk thermal conductivity alone. Under torch heating, polymer pyrolysis induces formation of a protective char layer, which contributes to thermal shielding by maintaining structural integrity and resisting erosion. Consistent with this behavior, surface morphology observations reveal accelerated contraction, pore enlargement, and fiber exposure in bare CF, ultimately leading to structural collapse, while CF/PA-6 exhibits limited surface damage owing to the presence of a polymer-derived char layer. As this char layer develops during repeated ablation cycles, the effective thermal resistance increases [43,44,45], resulting in delayed heat penetration into the composite interior. Consequently, CF/PA-6 demonstrates enhanced thermal insulation performance compared with CF alone (Figure 5a,b). Unlike highly graphitized carbon felts, which exhibit high carbon purity and excellent thermal stability due to their ordered carbon structure [46], the present work employs a highly porous rayon-based carbon fiber felt (~94% porosity). Raman analysis of the pristine carbon fiber felt (Figure S3) confirms that the material possesses a partially disordered (turbostratic) carbon structure. This porous architecture promotes uniform polymer distribution and supports stable char-layer development during flame exposure, indicating that thermal protection in this system primarily originates from dynamic microstructural evolution rather than intrinsic graphitic ordering. SEM images (Figure 5c–e) reveal distinct microstructural evolution across the ablation depth of CF/PA-6 after torch exposure. The outer ablation zone exhibits a highly porous and fragmented morphology, while the underlying pyrolysis zone consists of a continuous char structure conformably covering the carbon fiber bundles. The inner region maintains a relatively dense microstructure with minimal damage, indicating effective thermal shielding by the overlying layers. To further investigate pore structure evolution induced by torch exposure, nitrogen adsorption–desorption measurements were conducted for CF and CF/PA-6 samples before and after the ablation test (Figure S4 and Table S1). The pristine carbon fiber felt exhibited negligible measurable surface area, reflecting its open fibrous scaffold dominated by macroscopic voids rather than adsorption-accessible pores. After torch exposure, the CF sample showed only a small surface area of 1.10 m^2^ g^−1^ with a pore volume of 0.0032 cm^3^ g^−1^, indicating that bare carbon fiber does not develop a significant micro/mesoporous structure within the sensitivity of BET analysis. In contrast, the CF/PA-6 composite showed a pronounced increase in specific surface area from 1.77 to 35.72 m^2^ g^−1^ after ablation, accompanied by an increase in total pore volume from 0.00465 to 0.02174 cm^3^ g^−1^. The average pore diameter decreased from approximately 10.6 nm before ablation to 4.36 nm after torch exposure, suggesting the formation of finer mesoporous structures during polyimide pyrolysis. These results indicate that flame exposure induces the formation of a porous carbonaceous char network within the CF/PA composite. Such a heterogeneous char structure is consistent with the microstructural evolution observed in the SEM images and is expected to increase the tortuosity of heat-conduction pathways, thereby contributing to delayed heat penetration and improved thermal insulation behavior under repeated flame exposure.

Raman spectra were collected from representative regions across the ablation depth, including the outer ablation zone, the intermediate pyrolysis zone, and the relatively intact inner region, as identified from the cross-sectional SEM morphology. The Raman results (Figure 5f) show a non-monotonic variation in the I_G_/I_D_ ratio across the ablation depth, with values of 0.76, 1.37, and 0.83 for the outer, intermediate, and inner regions, respectively. The lower I_G_/I_D_ ratio in the outer region reflects severe oxidation and structural damage, leading to defect-rich disordered carbon. In contrast, the highest I_G_/I_D_ ratio observed in the intermediate pyrolysis zone indicates partial structural ordering associated with stabilized carbonization during torch exposure. In the inner region, the reduced I_G_/I_D_ ratio together with residual polymer-related features suggests limited thermal penetration and preservation of the underlying composite structure. FT-IR analysis (Figure 5g) further supports this chemical evolution, where the characteristic C–N stretching peak of polyimide becomes weakened in the pyrolysis zone and disappears in the ablated surface region. XPS analysis (Figure S5) provides complementary evidence for chemical transformation, revealing a transition from imide-containing polymer structures in the inner region to carbon-rich species near the ablated surface. Because the char layer formed during flame exposure remains largely disordered rather than fully graphitized, the structural evolution is more effectively interpreted through spectroscopic and morphological analyses rather than crystallographic methods. Although the increase in I_G_/I_D_ ratio indicates partial structural ordering during carbonization, SEM observations reveal that the resulting char layer remains structurally heterogeneous and fragmented rather than forming a dense graphitic structure. Such structural discontinuities interrupt continuous heat-conduction pathways and limit through-thickness heat transport. The formation of compositionally and structurally distinct zones, including an ablated outer layer, a stabilized pyrolysis region, and an intact interior, contributes to delayed heat penetration and improved backside thermal stability during repeated flame exposure. These experimental observations collectively suggest the formation of a hierarchical thermal-resistance structure, which is further summarized as a conceptual mechanism in Section 3.5.

### 3.5. Proposed Thermal Insulation Mechanism of CF/PA Composites

**Figure 6:**
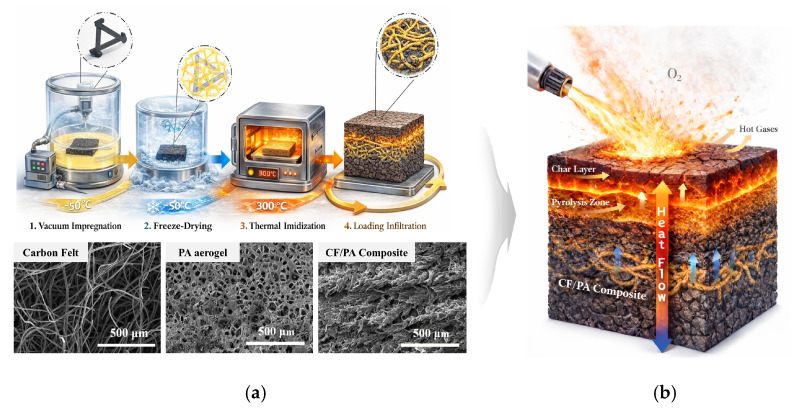
(**a**) Schematic illustration of the fabrication process of CF/PA composites and (**b**) the proposed ablation-protection mechanism during thermal exposure.

To provide a unified interpretation of the thermal responses observed in Section 3.3 and Section 3.4, a conceptual thermal-protection mechanism is proposed. CF/PA composites fabricated through the cyclic infiltration process (Figure 6a) exhibit a characteristic thermal response under flame exposure. Based on the combined results of thermal analysis, ablation testing, and microstructural characterization, a thermal insulation mechanism is proposed as illustrated in Figure 6b. Together with the underlying pyrolysis zone, this char layer forms a multilayer structure consisting of an outer ablation region, an intermediate carbonized layer, and an inner relatively intact composite region. This multilayer configuration plays a key role in heat attenuation during flame exposure. The porous char layer interrupts continuous heat-conduction pathways and acts as a diffusion barrier that limits oxygen ingress, thereby suppressing further oxidation of the carbon fiber network. Meanwhile, the intermediate pyrolysis zone provides a stabilized carbonized structure, while the inner region maintains its original composite integrity due to limited thermal penetration. As thermal exposure progresses, the cooperative interaction among these layers leads to progressively increased thermal resistance, delaying heat transfer toward the composite interior. Consequently, the effective thermal insulation behavior of CF/PA composites originates from dynamic microstructural evolution during ablation, where controlled pyrolysis and char-layer formation generate a gradient thermal-barrier structure that suppresses through-thickness heat transport under prolonged high-temperature exposure.

## 4. Conclusions

This study systematically investigated the structure–property relationships governing the thermal insulation behavior of carbon fiber felt/polyimide (CF/PA) composites fabricated via a freeze-drying-assisted multicycle impregnation process. Repeated impregnation promoted progressive densification, enhanced fiber–matrix interfacial bonding, and the formation of a continuous polyimide network within the porous carbon fiber framework. Under prolonged butane-torch exposure, the CF/PA composites exhibited markedly improved thermal protection performance compared with bare carbon fiber. While bare CF showed rapid backside temperature rise and severe structural degradation, CF/PA-6 demonstrated delayed heat penetration and maintained structural integrity even after extended high-temperature exposure. Cross-sectional analyses revealed the formation of a multilayer structure consisting of an ablated outer layer, a stabilized pyrolysis zone, and an intact inner region, which effectively suppressed internal erosion and through-thickness heat transfer. Although polyimide impregnation increased bulk density and room-temperature thermal conductivity, effective thermal protection under flame exposure was governed by dynamic microstructural evolution, including controlled polymer pyrolysis, char-layer formation, and multilayer thermal resistance development. Raman and FT-IR analyses further confirmed progressive carbonization and chemical stabilization within the pyrolysis zone, supporting the thermal shielding role of the polymer-derived char.

Overall, the enhanced thermal insulation performance of CF/PA composites originates from the synergistic effects of controlled pyrolysis, continuous char-layer formation, and hierarchical thermal-resistance architecture formed during exposure. These findings highlight the potential of multicycle-impregnated polymer–fiber composites as structurally robust and lightweight thermal protection materials for localized high-temperature environments. Future work may focus on optimizing precursor chemistry and pore architecture to further improve long-term durability and multifunctional thermal performance.

## Figures and Tables

**Table 1:** Comparison of the mass ablation rate of CF and CF/PA-6 after torch exposure under identical testing conditions. The ablation rate was calculated based on the mass loss before and after the thermal shock test. M_0_ represents the initial sample mass measured before flame exposure (t = 0 min).

		CF (Pure)			CF/PA-6		
		M_0_ (g)	M_1_ (g)	R_M_ (mg/s)	M_0_ (g)	M_1_ (g)	R_M_ (mg/s)
0 min		0.77 ± 0.014	-	-	1.46 ± 0.041	-	-
1 min			0.77 ± 0.014	0.06 ± 0.010		1.32 ± 0.037	2.26 ± 0.1765
3 min			0.76 ± 0.012	0.08 ± 0.027		1.30 ± 0.034	1.29 ± 0.0672
5 min			0.75 ± 0.010	0.14 ± 0.035		1.29 ± 0.027	1.44 ± 0.1197
7 min			0.75 ± 0.010	0.18 ± 0.027		1.28 ± 0.028	1.52 ± 0.1026
10 min			0.74 ± 0.010	0.17 ± 0.024		1.25 ± 0.017	1.17 ± 0.1401
15 min			0.73 ± 0.008	0.16 ± 0.026		1.22 ± 0.020	0.79 ± 0.0759
20 min			0.72 ± 0.006	0.17 ± 0.027		1.21 ± 0.018	0.84 ± 0.0965
25 min			0.70 ± 0.005	0.22 ± 0.031		1.20 ± 0.016	0.87 ± 0.0971
30 min			0.69 ± 0.004	0.25 ± 0.040		1.18 ± 0.013	0.95 ± 0.1158
35 min			0.68 ± 0.003	0.28 ± 0.050		1.17 ± 0.014	0.95 ± 0.1268
40 min			0.67 ± 0.004	0.32 ± 0.035		1.16 ± 0.011	1.00 ± 0.1231
45 min			0.66 ± 0.009	0.36 ± 0.020		1.15 ± 0.009	1.03 ± 0.1293
50 min			0.65 ± 0.013	0.40 ± 0.031		1.14 ± 0.007	1.07 ± 0.1371

M_0_: initial weight, M_1_: weight after ablation, R_M_: rate of weight loss.

## Data Availability

The original contributions presented in this study are included in the article/Supplementary Materials. Further inquiries can be directed to the corresponding authors.

## Supplementary Materials

The following supporting information can be downloaded at: https://www.mdpi.com/article/10.3390/polym18060742/s1, Figure S1: Flexural stress–strain curve of bare carbon-fiber felt (CF), showing deformation dominated by network rearrangement; Figure S2: Ablation test and the backside temperature evolution of CF and CF/PA-6 during repeated butane-torch ablation. (Inset; Front-surface changes after ablation cycles, showing severe erosion in CF and improved structural stability in CF/PA-6); Figure S3: (a) BET isotherm and (b) BJH pore distribution of CF and CF/PA-6 before and after torch ablation; Figure S4: Raman spectra of pristine CF; Figure S5: XPS C1s spectra of CF/PA composite at different depths after ablation: (a) SITE I (surface ablated zone), (b) SITE II (pyrolysis zone), and (c) SITE III (interior protected zone). Progressive loss of oxygen-containing peaks (C–O, C=O, O–C=O) and enhancement of graphitic C–C (sp^2^) indicate depth-dependent chemical stabilization and maturation of the char layer; Table S1: BET surface area and pore structure parameters of samples before and after torch ablation; Table S2: Comparison of evaluation scope adopted in representative phenolic-based thermal protection studies and the present CF/PA composite system. Unlike previous reports mainly focusing on thermal conductivity and ablation rate, this work additionally includes backside temperature monitoring, repeated flame exposure tests, and post-ablation structural integrity analysis. Refs. [29,47,48,49,50,51] are cited in Supplementary Materials.
