# Supplementary material for: Controlled Formation of Polyimide Aerogel Networks in Carbon Fiber Felt via Multicycle Freeze-Drying for Thermal Protection
Source: Polymers (Basel). 2026 Mar 18;18(6):742. doi: 10.3390/polym18060742 (PMC13029816; doi:10.3390/polym18060742)
Supplement: Supplementary file 1 [file polymers-18-00742-s001.zip › polymers-4161944-supplementary/polymers-4161944-supplementary.pdf]

Supplementary materials

Controlled Formation of Polyimide Aerogel Networks in Carbon Fiber Felt via Multicycle Freeze-Drying for Thermal Protection

Jae Won Lee[1,2], Han Kim[1], Yong-Ho Choa[2*], Sook Young Moon[1*]

[1]Institute of Advanced Composite Materials, Korea Institute of Science and Technology (KIST), Chudong-Ro 92, Bongdong-Eup, Wanju-Gun, Jeonbuk 55324, Republic of Korea

[2] Department of Materials Science and Chemical Engineering, Hanyang University, Ansan-si, Gyeongi-do 15588, Republic of Korea

*Corresponding author: choa15@hanyang.ac.kr; moon.sookyoung@kist.re.kr

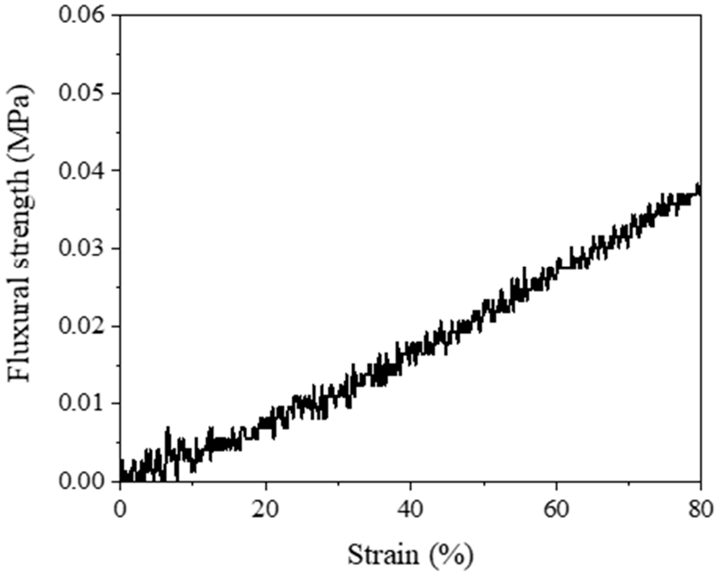

Figure S1. Flexural stress–strain curve of bare carbon-fiber felt (CF), showing deformation dominated by network rearrangement.

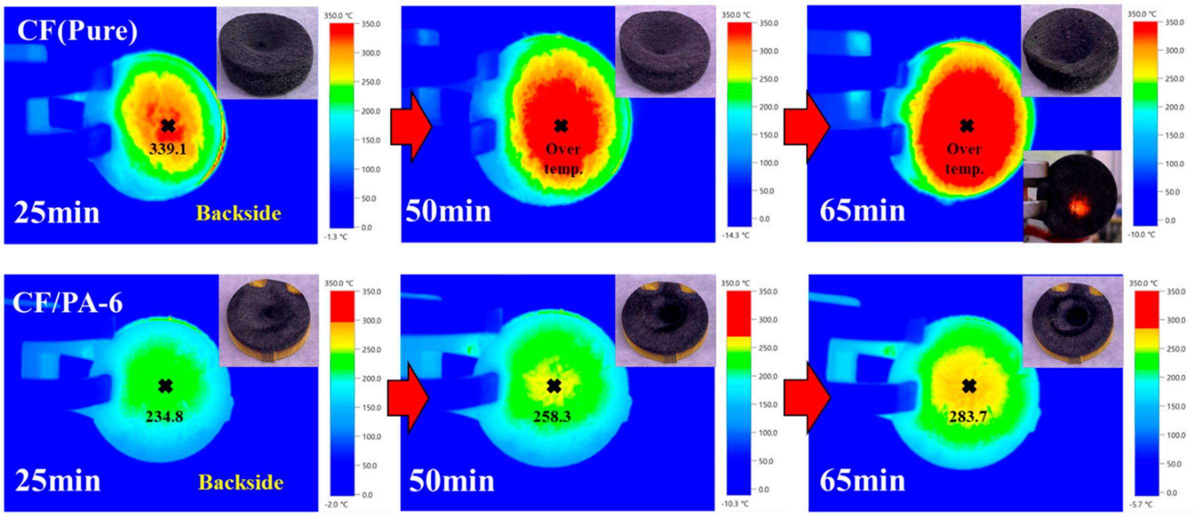

Figure S2. Ablation test and the backside temperature evolution of CF and CF/PA-6 during repeated butane-torch ablation. (Inset; Front-surface changes after ablation cycles, showing severe erosion in CF and improved structural stability in CF/PA-6)

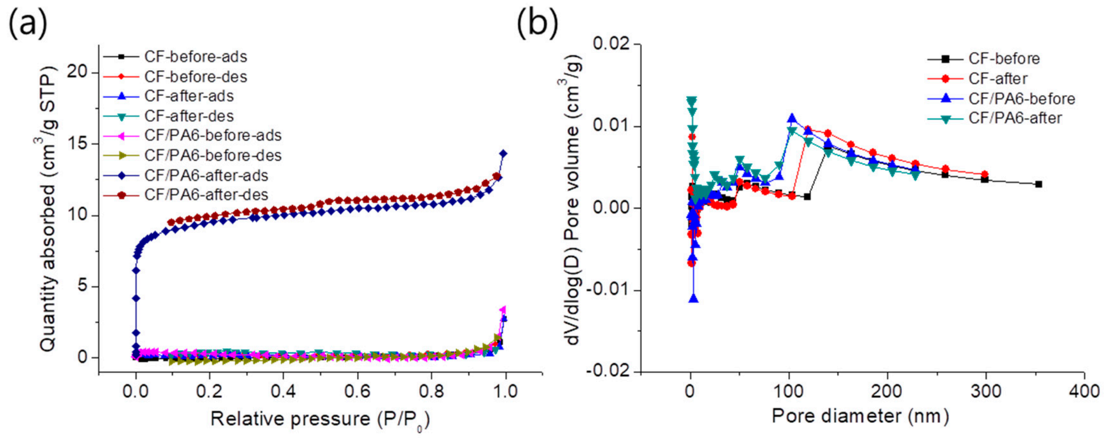

Figure S3. (a) BET isotherm and (b) BJH pore distribution of CF and CF/PA-6 before and after torch ablation.

Table S1. BET surface area and pore structure parameters of samples before and after torch ablation.

Sample	BET Surface Area (m^2/g)	BJH Pore volume (cm^3/g)	BJH pore diameter (nm)
CF-before	0.35	0.0028	560.79
CF-after	1.10	0.0032	14.66
CF/PA6-before	1.77	0.0046	10.59
CF/PA6-after	35.71	0.0217	4.36

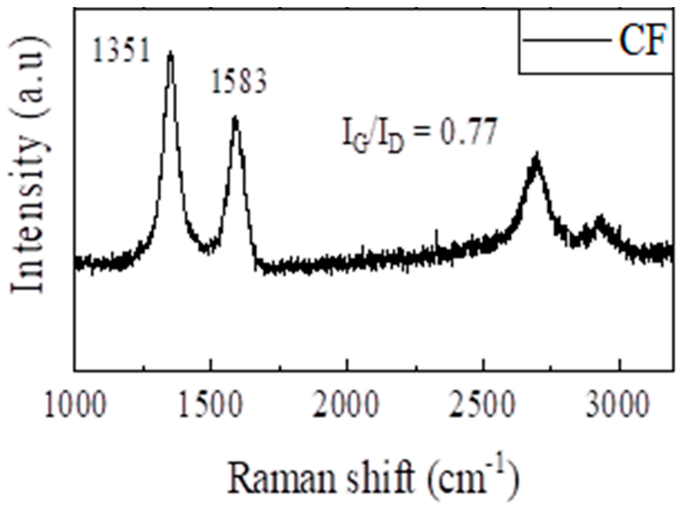

Figure S4. Raman spectra of pristine CF.

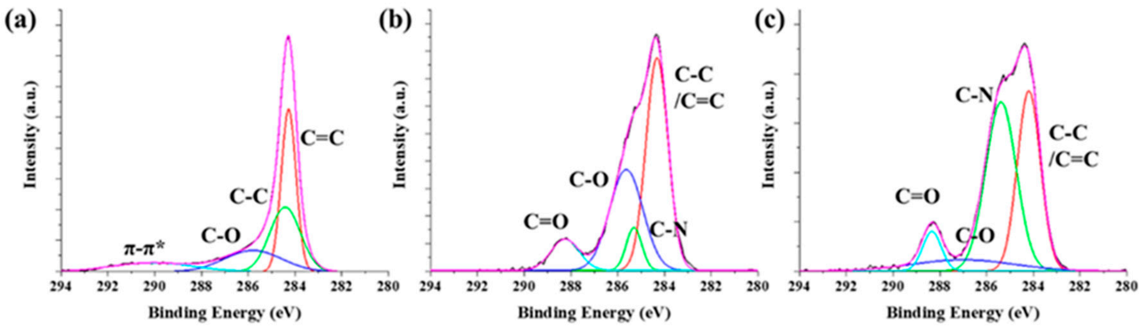

Figure S5. XPS C1s spectra of CF/PA composite at different depths after ablation: (a) SITE I (surface ablated zone), (b) SITE II (pyrolysis zone), and (c) SITE III (interior protected zone). Progressive loss of oxygen-containing peaks (C–O, C=O, O–C=O) and enhancement of graphitic C–C (sp^2) indicate depth-dependent chemical stabilization and maturation of the char layer.

Table S2. Comparison of evaluation scope adopted in representative phenolic-based thermal protection studies and the present CF/PA composite system. Unlike previous reports mainly focusing on thermal conductivity and ablation rate, this work additionally includes backside temperature monitoring, repeated flame exposure tests, and post-ablation structural integrity analysis.

Material system	Thermal conductivity measured	Ablation metric reported	Backside temperature monitoring	Repeated flame exposure test	Macroscopic shape retention after ablation	Cross-sectional analysis after ablation	Reference
Phenolic composite	O	O	X	X	O (Surface erosion described)	X	1
Polymer nanocomposite phenolic ablative	X	O	X	X	O (Char layer discussed)	X	2
Carbon fiber / silicone–phenolic composite (CFSP)	O	O	X	X	X	X	3
Carbon fiber / silicone–phenolic + aerogel	O	O	X	X	O (Porous structure discussed (micro-scale)	X	3
Phenolic–silica aerogel composite	O	O	X	X	O (Improved structural stability (qualitative))	X	4
Lightweight phenolic aerogel composite	O	O	X	X	X	X	5
Coupling-agent modified phenolic aerogel	O	X	X	X	X	X	6
CF/PA composites	O (Hot disk)	O (Ablation mass loss)	O	O (Repeated butane torch cycles)	O (Macro-level integrity retained)	O (Ablation / pyrolysis zones identified)	This work

References

1. Pelin, G.; Pelin, C, E.; Stefan, A.; Tsakiris, V.; Panait, A, A, M.; Costea, E. Oxy-Butane Ablation Testing of Thermal Protection Systems Based on Nanomodified Phenolic Resin Matrix Materials. *Polymers* **2023**, 15, 4016. https://doi.org/10.3390/polym15194016

2. Koo, J, H.; Wagner, K.; Pilato, L, A.; Wu, H. Polymer Nanocomposite Ablatives—Part III. *J. Compos. Sci.* **2025**, 9, 127. https://doi.org/10.3390/jcs9030127

3. Jin, X.; Xu, J.; Pan, Y.; Wang, H.; Ma, B.; Liu, F.; Yan, X.; Wu, C.; Huang, H.; Cheng, H.; Hong, C.; Zhang, X. Lightweight and multiscale needle quartz fiber felt reinforced siliconoxycarbide modified phenolic aerogel nanocomposite with enhanced mechanical, insulative and flame-resistant properties. *Compos. Sci. Technol.* **2022**, 217. https://doi.org/10.1016/j.compscitech.2021.109100

4. Wang, W.; Xu, W.; Jia, X.; Zhang, F.; Cao, Y.; Ma, C.; Wang, J.; Qiao, W.; Ling, L. A

facile in-situ strategy to fabricate lightweight carbon fiber/silicone-phenolic aerogel composites with superior toughness and ablation performance. *J. Mater. Sci.* **2024**, 59, 18488-18498. https://doi.org/10.1007/s10853-024-10282-7

5. Wang, Z.; Kong, Y.; Nie, M.; Liu, K.; Liu, Q.; Shen, X. Hydrophobic phenolic/silica aerogel composites with high fire safety and strength for efficient thermal insulation. *RSC adv.* **2025**, 15, 43095. https://doi.org/10.1039/d5ra07166f

6. Ye, D.; Lv, H.; Zheng, Z.; Luo, L. Preparation and Properties of Flexible Phenolic Silicone Hybrid Aerogels for Thermal Insulation. *Molecules* **2024**, 29, 4942. https://doi.org/10.3390/molecules29204942
